# Metabolic Networks of *Sodalis glossinidius*: A Systems Biology Approach to Reductive Evolution

**DOI:** 10.1371/journal.pone.0030652

**Published:** 2012-01-24

**Authors:** Eugeni Belda, Francisco J. Silva, Juli Peretó, Andrés Moya

**Affiliations:** 1 Institut Cavanilles de Biodiversitat i Biologia Evolutiva, Universitat de València, València, Spain; 2 Departament de Genètica, Universitat de València, València, Spain; 3 Unidad Mixta de Investigación de Genómica y Salud (Centro Superior de Investigación en Salud Pública, CSISP/Institut Cavanilles), Universitat de València, València, Spain; 4 Departament de Bioquímica i Biologia Molecular, Universitat de València, València, Spain; The Centre for Research and Technology, Hellas, Greece

## Abstract

**Background:**

Genome reduction is a common evolutionary process affecting bacterial lineages that establish symbiotic or pathogenic associations with eukaryotic hosts. Such associations yield highly reduced genomes with greatly streamlined metabolic abilities shaped by the type of ecological association with the host. *Sodalis glossinidius*, the secondary endosymbiont of tsetse flies, represents one of the few complete genomes available of a bacterium at the initial stages of this process. In the present study, genome reduction is studied from a systems biology perspective through the reconstruction and functional analysis of genome-scale metabolic networks of *S. glossinidius*.

**Results:**

The functional profile of ancestral and extant metabolic networks sheds light on the evolutionary events underlying transition to a host-dependent lifestyle. Meanwhile, reductive evolution simulations on the extant metabolic network can predict possible future evolution of *S. glossinidius* in the context of genome reduction. Finally, knockout simulations in different metabolic systems reveal a gradual decrease in network robustness to different mutational events for bacterial endosymbionts at different stages of the symbiotic association.

**Conclusions:**

Stoichiometric analysis reveals few gene inactivation events whose effects on the functionality of *S. glossinidius* metabolic systems are drastic enough to account for the ecological transition from a free-living to host-dependent lifestyle. The decrease in network robustness across different metabolic systems may be associated with the progressive integration in the more stable environment provided by the insect host. Finally, reductive evolution simulations reveal the strong influence that external conditions exert on the evolvability of metabolic systems.

## Introduction

Genome reduction is a common evolutionary process observed in bacterial lineages that have established associations, both symbiotic and pathogenic, with eukaryotic hosts. In advanced stages of the process one finds highly streamlined genomes and minimal metabolic systems unable to function outside their hosts [1]. This process has been extensively characterized in bacterial endosymbionts of insects, where nutritional associations with bacterial endosymbionts allow insects to colonize novel ecological niches with unbalanced nutritional sources [2], [3]. This is due to drastic changes in the population structure and selective pressures associated with the evolutionary transition from a free-living to a host-dependent lifestyle. During this transition, inactivating mutations are accumulated over non-essential genes leading to a massive accumulation of pseudogenes throughout the bacterial chromosome [4]. Characteristically, there is also a massive proliferation of different types of mobile genetic elements in these initial stages of genome reduction, representing an important source of genome rearrangements [5], [6]. This gene inactivation process is enhanced by drastic reduction in the effective population size of these bacterial endosymbionts (i.e. population bottlenecks associated to their strict vertical transmission from mothers to offspring), allowing the accumulation of slight deleterious mutations by random genetic drift in a process known as Muller's ratchet [7]. This large amount of non-functional DNA is subsequently lost in long-term bacterial endosymbionts through a stepwise process, involving many small and some large deletion events [8], [9], [10], [11]. This process eventually leads to very small bacterial genome sizes such as those of the aphid endosymbiont *Buchnera aphidicola* [12], [13], [14], [15], the ant endosymbiont *Blochmannia* [16], [17], or the psyllid endosymbiont *Carsonella rudii* [18], [19]. The dynamics of gene loss in long-term symbiotic associations can be studied by comparative genomics [20], [21], [10]. However, in ancient endosymbiont-insect host associations, it is difficult to determine the evolutionary events triggering the initial transition to a host-dependent lifestyle, or the point at which the free-living ancestor lost the extracellular replicative stage.

*S. glossinidius*, the secondary endosymbiont of tsetse flies, represents one of the few available complete genomes of a bacterium at the initial stages of symbiosis [22]. Tsetse flies also harbour the obligatory mutualistic bacteria *Wigglesworthia glossinidia*. The latter has an ancient association with the tsetse host, having a highly streamlined genome (698 kb) that mostly retains cofactor biosynthetic pathways responsible for supplying the host with vitamins lacking in vertebrate blood, its sole nutrient source [23]. By contrast, *S. glossinidius* represents a much more recent symbiotic association. This is revealed by the fact *S. glossinidius* can be cultured under laboratory conditions, as can other recent bacterial endosymbionts, like secondary endosymbiont of hippoboscid louse flies [24]. Moreover, its genome size is 4.2 megabases, closer to a free-living bacterium like *Escherichia coli*, without any compositional bias [22], [25], [26]. However, the most important feature of this genome is its extremely low coding density, consequence of a massive process of gene inactivation, with 972 pseudogenes described in the original genome annotation and extended to 1501 pseudogenes in a recent re-annotation [27]. This makes *S. glossinidius* an ideal model system to study the complete genome reduction process covering both the initial transition from free-living to host-dependent lifestyle and the reductive evolution towards minimal metabolic systems associated to long-term symbiotic associations. This is because its current gene content, with the whole set of genes and pseudogenes, represent a direct hallmark of the ancestral gene content of the bacteria before the ecological transition to host-dependent lifestyle, after which the changes in selective pressures and population dynamics of the bacteria generates the massive accumulation of pseudogenes observed in the actual genome. Furthermore, predictions can be made as to how the current system could evolve further within the context of a reductive evolutionary process. This can be analyzed through the reconstruction of *S. glossinidius* metabolic networks at different stages of the genome reduction process and through functional analysis by Flux Balance Analysis (FBA). FBA enables quantitative assessment of the function of metabolic systems by finding the optimal distributions of metabolic fluxes across reactions of the network that optimize a particular objective function, normally defined through a biomass equation. Biomass production maximization is equivalent to experimentally determined cellular growth phenotypes, as demonstrated in model organisms like *E. coli* K12 [28], [29].

Available genome-scale metabolic networks range from important model organisms to pathogens and bacterial species of biotechnological interest [30]. Recently, a genome-scale metabolic network of *B. aphidicola* from the pea aphid *Acyrthosiphon pisum* was published, revealing a highly streamlined functional profile and high fragility [31]. In the context of bacterial evolution, FBA of the *E. coli* K12 metabolic network, combined with comparative genomics, have revealed the predominant role of horizontal gene transfer over gene duplication in the recent evolution of *E. coli* K12, with horizontally transferred genes playing an essential role under specific external conditions [32], [33]. More specifically related with the reductive genome evolutionary process, the gene content of real minimal genomes, like in *B. aphidicola* and *W. glossinidia*, can be accurately predicted by successive gene deletions in the *E. coli* K12 metabolic network. This is possible because external conditions mimic the nutrient availability within the insect host corresponding to each bacterium [34]. Recently, the work of Pal et al. has been extended to predict dynamic aspects of the gene loss process in the *B. aphidicola* lineage [35], in an effort to predict how much of the gene loss process in this ancient bacterial endosymbiont can be accounted for by metabolic constraints. However, although these approaches provide novel insights into the dynamics and final stages of genome reduction in endosymbiotic bacteria that have long-term associations with their insect host, there is a critical issue that remains unanswered: What are the evolutionary events that determine the initial change from the free-living to the endosymbiotic state?

Gene loss dynamics can be studied by comparative genomics in many of these ancient symbiotic lineages from the point of divergence from their hypothetical free-living ancestor [10], [36], [37]. However, such approaches cannot quantitatively evaluate which of these gene loss events had the greatest impact on the overall functionality of the system and, consequently, on the loss of the free-living state triggering the genome reduction process. In this context, genome-scale metabolic modelling of a bacterium that has recently adopted a host-dependent lifestyle, like *S. glossinidius*, can quantitatively assess the gene loss events in a system for which the ancestral genome can be reliably inferred from the whole set of genes and pseudogenes.

The present work reports the reconstruction of genome-scale metabolic networks of *S. glossinidius* at different stages of the genome reduction process, in order to study its reductive evolution from a systems biology perspective. Metabolic phenotypes of ancestral and extant metabolic networks were quantitatively assessed by FBA, to determine the evolutionary events that could account for the transition from a free-living to a host-dependent lifestyle. Finally, the robustness of these metabolic systems to gene deletion events was compared to analyze how the transition to a host-dependent lifestyle correlates with the ability of a metabolic system to adapt to deletion changes.

## Results

### Reconstruction of *S. glossinidius* metabolic networks

The ancestral metabolic network of *S. glossinidius* comprised 668 gene products, 741 internal reactions, and 690 metabolites, of which 547 metabolites were cytoplasmic and 143 were extracellular (File S1). This network contained not only functional genes but also pseudogenization and gene deletion events taking place during *S. glossinidius* evolution from its free-living ancestor. Gene deletion events had been restricted to those genes presents in closer relative enterobacterial genomes and for which the genomic context is extensively conserved between *S. glossinidius* and *E. coli* K12, in order to exclude deleted genes originated by horizontal gene transfer events that may not be functional in the ancestor. Of the 668 gene products, 479 corresponded to functional genes, 148 to pseudogenes, and 41 to genes deleted during the evolution of *S. glossinidius* from its free-living ancestor. The 627 CDSs represented 16% of the total number of CDSs (genes and pseudogenes) in the genome, which was in the range of other genome-scale network reconstructions (see Table 1). Out of the 741 internal reactions included in the network, 683 reactions (92.2%) had at least one assigned gene, pseudogene or deleted gene. This ancestral metabolic network included 27 pseudogenes without sequence similarity with *E. coli* K12 and with specific metabolic functions in terms of reaction stoichiometry based on the results of functional re-annotation of the genome. Most of these pseudogenes encode isozymes of functional enzymatic activities although they also include seven reactions not present in *E. coli* K12 iJR904, representing functional abilities of the *S. glossinidius* ancestral metabolism (see Table S1).

**Table 1:** Comparison of the genome characteristics and *in silico* metabolic networks of *S. glossinidius*, *B. aphidicola* APS and *E. coli* K12.

	*S. glossinidius* ancestor	*S. glossinidius*	*E. coli* K12	*B. aphidicola* Bap
Genome characteristics				
Genome length (bp)	4,171,146	4,171,146	4,639,675	640,681
GC content (%)	54	54	50	26
CDSs	2,431 (genes) +1,501 (pseudogenes)	2,431 (genes) +1,501 (pseudogenes)	4,144	564
*In silico* metabolic network characteristics				
Number of reactions	741	560	931	210
Reactions with CDSs (%)	92.2	90	94	96
Number of CDSs	668 (479 genes+148 pseudogenes+41 deleted genes)	458	904	196
% genome	16	18.8	21.8	34.2
Metabolites	547	481	625	240

The extant metabolic network was reconstructed by removing all reactions catalyzed by the 148 pseudogenes and the 41 deleted genes from the ancestral network. This rendered an extant network composed of 458 gene products, 560 internal reactions including transport processes, cytoplasmic reactions and biomass equation, and 624 metabolites, of which 481 were cytoplasmic and 143 were extracellular (see Table 1, File S2). Of the 560 internal reactions, 502 had at least one gene assigned (90%). The 458 genes in the extant network represented 18.8% of *S. glossinidius* genes. In the transition to the extant network, 21 functional genes were removed due to their inclusion in enzymatic complexes where at least one of the components was pseudogenized or deleted. In addition, 13 functional genes, without sequence similarity with *E. coli* K12 by OrthoMCL, were incorporated to both ancestral and extant networks of *S. glossinidius* based on the functional re-annotation of the genome [27] (see Table S2).

### Analysis of *S. glossinidius* metabolic networks by FBA: Transition to the host-dependent lifestyle

**Figure 1:**
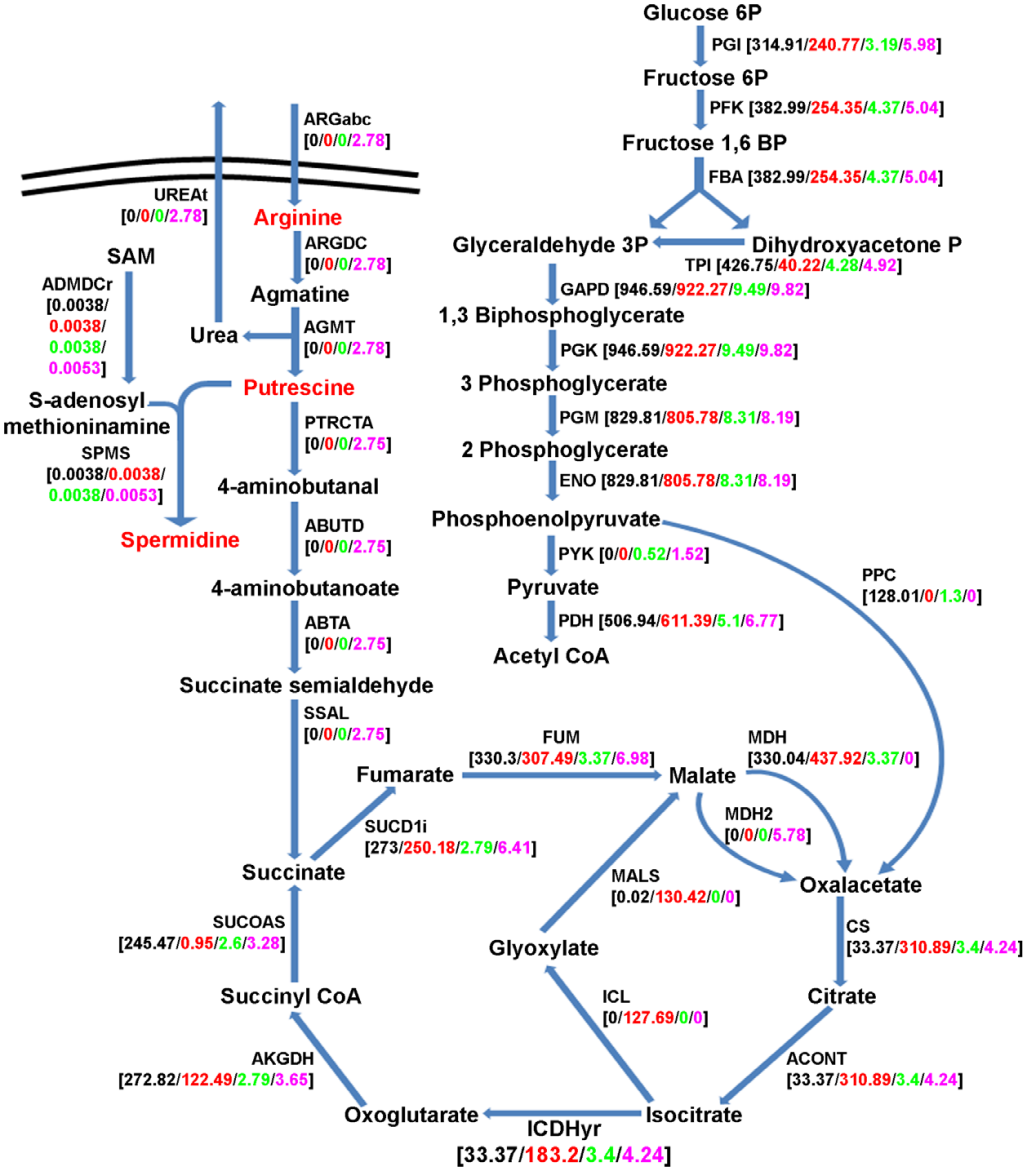
Phosphoenolpyruvate carboxylase (PPC reaction) inactivation effect in *E. coli* K12 iJR904 and *S. glossinidius* metabolic networks. Reactions of glycolysis, TCA cycle, and transport and metabolization of external L-arginine are represented together with their corresponding reaction fluxes in FBA simulations. Red metabolites are included in the biomass equation. Black values correspond to reaction fluxes in the *E. coli* K12 iJR904 network with glucose as sole external carbon source. Red values correspond to reaction fluxes in the *E. coli* K12 iJR904 network under the same conditions but removing the PPC reaction. Green values correspond to reaction fluxes in the *S. glossinidius* ancestral network, with glucose as sole external carbon source. Purple values correspond to reaction fluxes in the *S. glossinidius* extant network with glucose and L-arginine as external metabolites. Reaction fluxes are represented in mmol gr DryWeight^−1^ hr^−1^.

Functionality of both metabolic networks of *S. glossinidius* (ancestral and extant) was analyzed by FBA and compared with the functional profile of a free-living relative, like *E. coli* K12. As objective function, the biomass equation defined for the *E. coli* K12 iJR904 network [38] was included in both ancestral and extant networks of *S. glossinidius*. This biomass equation reflects the basic requirements in terms of essential metabolites (amino acids, nucleotides, phospholipids, cofactors) ensuring cell survival. The recent transition of *S. glossinidius* to a host-dependent lifestyle can be tested by evaluating the functionality of its ancestral and functional metabolic networks in a minimal aerobic environment with glucose as sole external carbon source for biomass production. This was done by fixing the lower bounds of their corresponding exchange reactions to −6 mmol gr DW^−1^ hr^−1^ for glucose and to −20 mmol gr DW^−1^ hr^−1^ for molecular oxygen (O_2_), as described in the *E. coli* K12 iJR904 metabolic network [38]. With glucose as sole external carbon source, the ancestral metabolic network of *S. glossinidius* showed a biomass production rate of 0.545 gr DW (mmol Glucose)^−1^, very similar to the biomass yield of the *E. coli* K12 iJR904 network (0.5391 gr DW (mmol Glucose)^−1^). By contrast, the *S. glossinidius* extant network was completely non-functional under these minimal conditions. This was due to two main gene inactivation events affecting biosynthetic pathways and a single pseudogenization event affecting an essential enzyme activity of the central metabolism, occurring during *S. glossinidius* evolution. First, the pseudogenization of genes *glgA* and *glgB*, encoding glucose 1-phosphate adenylyltransferase and glycogen synthase respectively, impeded glycogen biosynthesis from ATP and α-D-glucose 1-phosphate. Glycogen was a component of biomass equation, and consequently these two pseudogenization events render a lethal phenotype in terms of biomass production in the *S. glossinidius* extant network. The elimination of glycogen from the biomass equation solved this problem and rendered a functional phenotype in terms of biomass production. Second, the pseudogenization of genes *argA*, *argG*, *argD* and *argC* prevented L-arginine biosynthesis from L-glutamate, as recently described in the re-annotation of the genome [27]. L-arginine was also a biomass constituent, but unlike glycogen, its removal from the biomass equation still rendered a lethal phenotype in terms of biomass production. This was because the inactivation of the L-arginine biosynthesis pathway prevented both putrescine and spermidine biosynthesis. These two polyamines can only be synthesized from L-arginine (see Figure 1) due to pseudogenization of the *speC* gene, which encodes an L-ornithine decarboxylase (ps_SGL1267c) catalyzing single-step decarboxylation of L-ornithine to putrescine [39]. A viable phenotype in terms of biomass production was obtained for the *S. glossinidius* extant network by incorporating an external source of L-arginine (0.7509 gr DW (mmol Glucose)^−1^).

In addition to these pseudogenization events affecting biosynthetic pathways, the pseudogenization of the gene *ppc* (ps_SGL1383), encoding the enzyme phosphoenolpyruvate carboxylase (PEP carboxylase), had particularly lethal effects on the overall metabolic system. This key enzymatic activity catalyzes the anaplerotic reaction of carboxylation of the glycolytic intermediate phosphoenolphyruvate to the TCA cycle intermediate oxalacetate [40]. This event was analyzed by comparing the reaction fluxes of *E. coli* K12 iJR904 and *S. glossinidius* metabolic networks on reactions of the central metabolism and L-arginine metabolism (see Figure 1). With glucose as sole external carbon source, in both the *E. coli* K12 iJR904 and *S. glossinidius* ancestral networks, reaction fluxes were distributed throughout the glycolysis and TCA cycle. Meanwhile, oxalacetate was replenished though PEP carboxylase, and putrescine synthesized by decarboxylation of the L-arginine intermediate L-ornithine. Removing the PEP carboxylase reaction from the *E. coli* K12 iJR904 metabolic network activated the glyoxylate bypass, a second anaplerotic pathway, coinciding with experimental results obtained measuring reaction fluxes of *E. coli* K12 *ppc* mutants [41], [42]. However, in the *S. glossinidius* genome, there was no sign of genes or pseudogenes encoding enzymes of the glyoxylate bypass, and, consequently, removal of the PEP carboxylase reaction alone from the *S. glossinidius* ancestral network drastically reduced biomass production of the whole metabolic system to 1.5077× e^−14^ gr DW (mmol Glucose)^−1^. Under these conditions, the supply of exogenous L-arginine was essential for the *S. glossinidius* functional metabolic system. This was due not only to its role as biomass constituent and precursor of additional biomass constituents, such as putrescine and spermidine, but also because putrescine must be broken down to TCA cycle intermediate succinate to overcome the absence of PEP carboxylase (see Figure 1).

### Robustness analysis in *S. glossinidius* and *E. coli* K12 iJR904 metabolic networks

**Figure 2:**
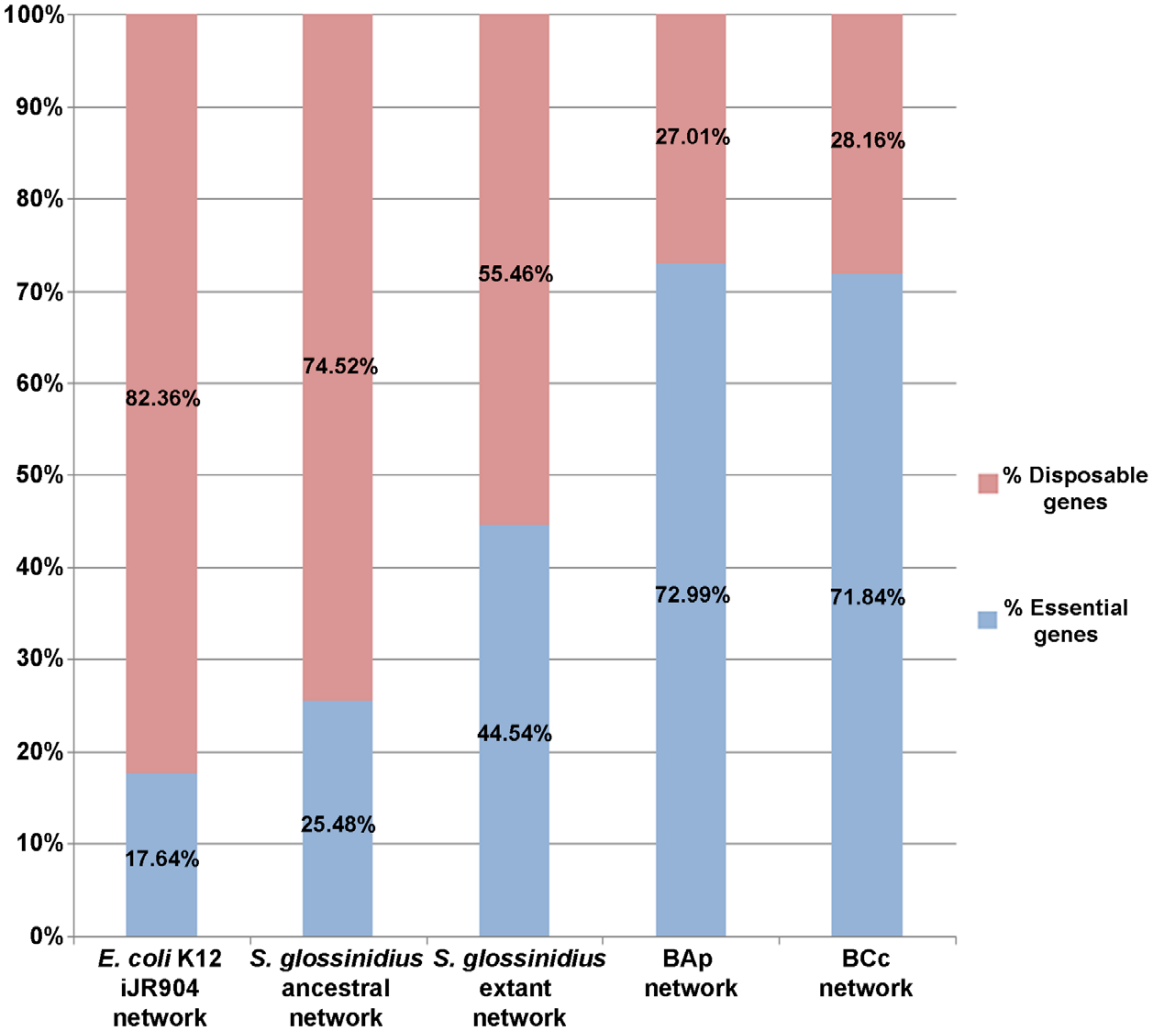
Robustness analysis on *E. coli* K12, *S. glossinidius* and *B. aphidicola* BAp and *B. aphidicola* BCc metabolic networks. The fraction of essential and non-essential genes in single knockout simulations on different genome-scale metabolic networks is represented. Essential genes are defined as those genes whose deletion results in a decrease of more than 99% of the original biomass production.

The genetic robustness of *E. coli* K12 iJR904 and *S. glossinidius* metabolic networks to gene deletion events was also analyzed. The results of this analysis revealed that under aerobic conditions with glucose and L-arginine as external metabolites, the *S. glossinidius* ancestral network behaved similarly to that of *E. coli* K12 iJR904 in the fraction of essential genes, or genes whose deletion renders a lethal phenotype in terms of biomass production. Single knockout simulations revealed 160 and 166 genes in the *E. coli* K12 iJR904 and *S. glossinidius* ancestral networks, respectively, whose deletion rendered a biomass production rate of 0 gr DW (mmol Glucose)^−1^ in FBA simulations. This number increased to 204 essential genes in the *S. glossinidius* extant network, reflecting a decrease in network robustness to gene deletion events that can be put down to the gene inactivation process. Comparison of robustness to double gene deletion events over the three metabolic systems to identify synthetic lethal pairs, whose simultaneous deletion prohibits growth, gave similar results [43]. To avoid the masking effect of essential genes, this analysis was restricted to gene pairs for which individual gene deletions render non lethal phenotypes in terms of biomass production. Results revealed an increase in the number of synthetic lethal pairs from *E. coli* K12 iJR904 (60 synthetic lethal pairs) to the *S. glossinidius* ancestral network (113 synthetic lethal pairs) and to the *S. glossinidius* extant network (211 synthetic lethal pairs). To determine whether this decrease in network robustness extended to bacterial endosymbionts at more advanced stages of the genome reduction process, the same single and double knockout simulations were carried out on the metabolic networks of two *B. aphidicola* strains: BAp (from the pea aphid *A. pisum*) [31] and BCc (from the aphid *Cinara cedri*). BCc was reconstructed from *B. aphidicola* BAp by removing genes and reactions absent in the more reduced genome of *B. aphidicola* BCc and adjusting the biomass equation composition. Figure 2 shows the results of single knockout simulations. If we consider an essential gene to be one whose deletion decreases original biomass production by over 99%, the fraction of essential genes increased progressively from a free-living bacteria, like *E. coli* K12 (17.6% of network genes), to a bacterial endosymbiont in the initial stages of transition to a host-dependent lifestyle, like *S. glossinidius* extant network (44.5% of network genes). This fraction increases to 73% and 71.8% of network genes in the metabolic networks of an ancient bacterial endosymbiont with a highly streamlined genome like *B. aphidicola* BAps and BCc, respectively. Double gene deletion simulations with *B. aphidicola* metabolic networks rendered 57 lethal double knockouts in BAps and one lethal double knockout in BCc, which contrasts with the results obtained for *S. glossinidius* and *E. coli* K12 iJR904 metabolic networks. However, this can be explained by the reduced number of non-essential genes included in the analysis of BAps (57 genes) and BCc (29 genes) compared with *E. coli* K12 iJR904 (747 genes), *S. glossinidius* ancestral (462 genes) and *S. glossidius* extant networks (254 genes). Also accountable, are the differences in external conditions and biomass composition between *B. aphidicola* metabolic networks and *E. coli* K12-*S. glossinidius* metabolic networks. Overall, these results revealed a common trend in the reductive evolutionary process with a progressive decrease in the robustness of metabolic systems to gene deletion events associated with the transition to a host-dependent lifestyle.

### Reductive evolution simulations on the functional metabolic network of *S. glossinidius*

**Figure 3:**
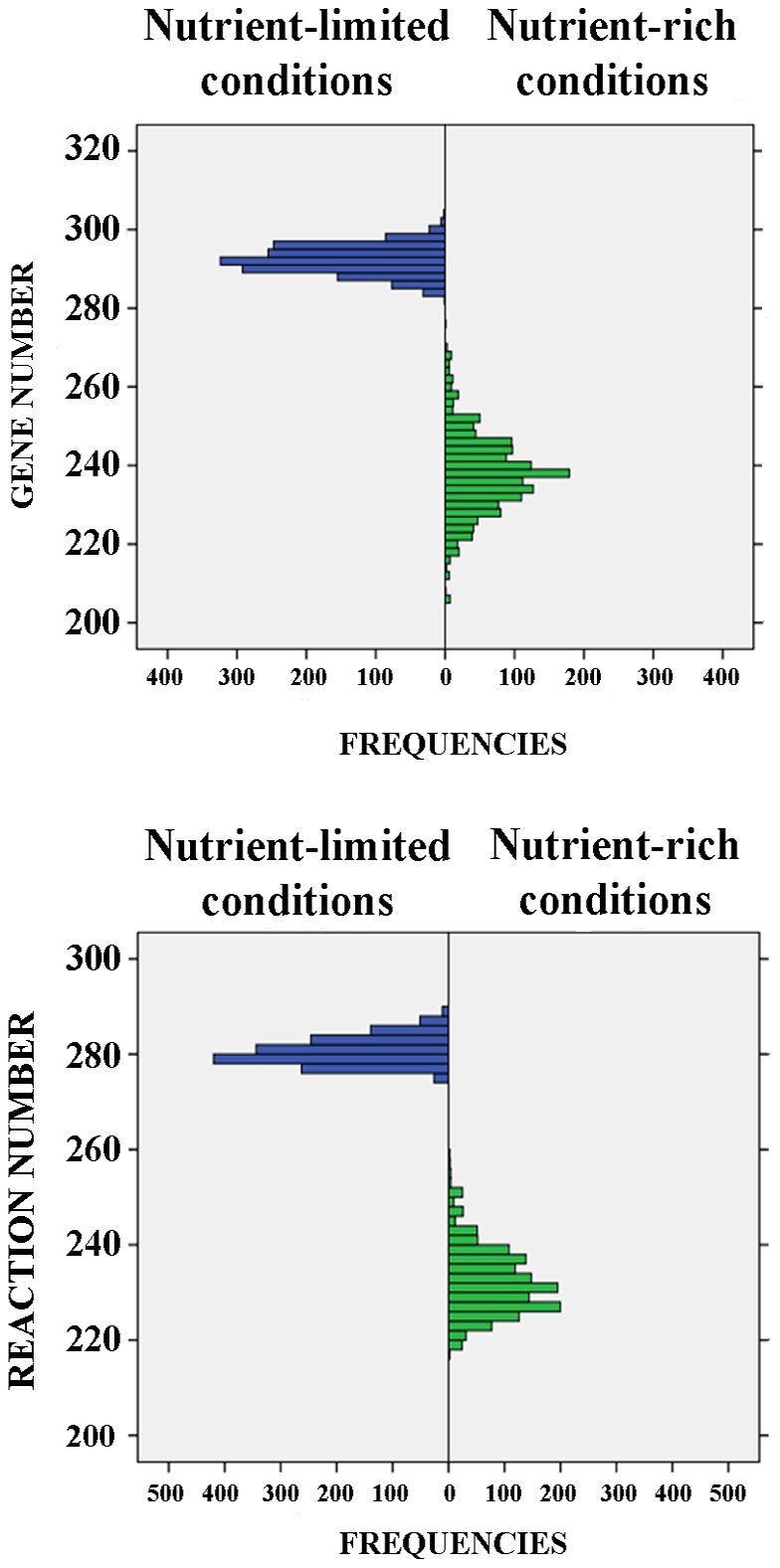
Gene and reaction number distribution in reductive evolution simulations. Distribution of the number of genes and reactions in 1500 minimal networks under nutrient-rich and nutrient-limited conditions.

To analyze the possible evolutionary outcomes of genome reduction in the *S. glossinidius* functional metabolic network, reductive evolution simulations were carried out, as described in Material and Methods. Results are shown in Table 2. Under nutrient-limited conditions, an average network size of 280.11 internal reactions and 291.75 genes was obtained for the 1500 resulting minimal networks. In addition, 84.6% of the reactions and 87.4% of the genes were shared by all minimal networks. In comparison, minimal networks under nutrient-rich conditions showed an average network size of 231.9 internal reactions and 237.25 genes in the total set of 1500 minimal networks obtained, of which 60.9% of the reactions and 58.7% of the genes were common to all minimal networks. These results pointed to a higher degree of plasticity, in terms of both gene and reaction content, in minimal networks evolved under a nutrient-rich environment compared with those evolved under a minimal aerobic medium with only glucose and L-arginine as external metabolites. In the latter scenario, in terms of gene and reaction content, the range of potential outcomes of reductive evolution simulations were further restricted by the genome reduction process (see Figure 3).

**Table 2:** Results of reductive evolution simulations under nutrient-limited and nutrient-rich conditions.

		10% original biomass	5% original biomass	1% original biomass	Total 1500 minimal networks
Reductive evolution simulations nutrient-limited conditions	Mean gene number(*1)	291.47 (±0.17)	292.15 (±0.16)	291.64 (±0.14)	291.75 (±0.09)
	Mean reaction number(*1)	280.03 (±0.12)	280.51 (±0.13)	279.78 (±0.12)	280.11 (±0.07)
	Common genes 500 minimal networks	258	257	261	255
	Absent genes 500 minimal networks	129	128	127	127
	Common reactions 500 minimal networks	240	239	242	237
	Absent reactions 500 minimal networks	208	205	203	200
Reductive evolution simulations nutrient-rich conditions	Mean gene number(*1)	237.99 (±0.45)	236.91 (±0.46)	236.84 (±0.45)	237.25 (±0.26)
	Mean reaction number(*1)	232.03 (±0.30)	231.91 (±0.32)	231.74 (±0.32)	231.9 (±0.18)
	Common genes 500 minimal networks	139	139	139	139
	Absent genes 500 minimal networks	114	111	112	111
	Common reactions 500 minimal networks	141	141	141	141
	Absent reactions 500 minimal networks	191	185	186	185

*1The mean gene and reaction number of minimal networks is shown with the corresponding standard error (in brackets).

The gene and reaction content of minimal networks obtained under both external conditions were also compared. This analysis showed that 138 out of 139 essential genes under nutrient-rich conditions were also essential under nutrient-limited conditions, whereas all 141 essential reactions under nutrient-rich conditions were also essential under nutrient-limited conditions. These results coincided with theoretical expectations, in that a reaction or a gene essential for system viability under a nutrient-rich environment was also expected to be essential in a more restricted minimal environment with glucose and L-arginine as sole external metabolites. The only gene always present in all minimal networks under nutrient-rich conditions, but not under nutrient-limited conditions, was SG0465, coding for an aromatic amino acid permease (AroP), responsible for transporting the biomass constituents histidine, phenylalanine, tyrosine and tryptophan in both *E. coli* K12 iJR904 and *S. glossinidius* networks. The removal of any reactions involved in these amino acids' biosynthesis makes AroP an essential gene in a nutrient-rich environment. Table S3 lists the whole set of essential genes and reactions, under all conditions. These include the complete biosynthetic pathways of biomass constituents that cannot be assimilated from external sources in a nutrient-rich environment, like the complete pathways for putrescine and spermidine biosynthesis from exogenous L-arginine. Also included are the L-arginine ABC transport system, the complete biosynthetic pathways for membrane phospholipids (phosphatidylglycerol, phosphatidylethanolamine and cardiolipin), peptidoglycan and the bacterial lipopolysaccharide, as well as the complete pathways for the biosynthesis of different cofactors, like methylenetetrahydrofolate, coenzyme A, FAD or NAD. There were 117 genes and 96 reactions that seemed essential in a nutrient-limited environment but not in a nutrient-rich environment (conditionally essential). Therefore, these could be lost in the future reductive evolution of *S. glossinidius* in the context of its ecological association with the tsetse host (see Table S4). In fact, many of these genes and reactions were involved in amino acid biosynthesis, which is the most streamlined functional class in the *W. glossinidia* genome, the primary endosymbiont of tsetse flies in more advanced stages of the genome reduction process [23]. These included the complete biosynthetic pathways for asparagine, aspartate, alanine, cysteine, serine, glycine, histidine, threonine, methionine, lysine, tyrosine, tryptophan, phenylalanine, valine, leucine, and isoleucine. For all these amino acids, a functional transport system was retained in the *S. glossinidius* extant network, allowing their exogenous assimilation in a nutrient-rich environment. In the case of lysine biosynthesis, only the gene *lysA* (SG1988), encoding a diaminopimelate decarboxylase responsible for the final decarboxylation of meso-diaminopimelate to lysine, was included as a conditionally essential gene. The remaining genes and reactions for meso-diaminopimelate biosynthesis from L-aspartate were essential under all conditions. This can be explained by the key role of meso-diaminopimelate as precursor of peptidoglycan, which is a biomass constituent, coinciding with the experimental assessments of gene essentiality in *E. coli* K12 by transposon mutagenesis [44]. Finally, 109 genes and 172 reactions proved non-essential or absent in all minimal networks under both original and nutrient-rich conditions (see Table S5). These included the remaining steps of metabolic pathways in which some components have been pseudogenized or deleted in the ancestral network, like the remaining steps of L-arginine and thiamine biosynthetic pathways, both pseudogenized in *S. glossinidius* [26], [27]. If fact, many of these genes could be selectively neutral genes in the sense of intact genes that are non-functional in the new host-associated environment but that has not yet accumulated mutations that allows them to identified as pseudogenes, as has been postulated for secondary symbiont of aphids *Serratia symbiotica* [45]. Also there were complete biosynthetic pathways for metabolites not included in the biomass equation, such as the genes involved in ubiquinone, biotin, pyridoxine 5-phosphate and heme group biosyntheses.

## Discussion

The present work analyzes the genome reduction process from a systems biology perspective. To do so, we have reconstructed and functionally analyzed genome-scale metabolic networks of the secondary endosymbiont of tsetse flies *S. glossinidius* at different evolutionary stages. The functionality of ancestral and functional metabolic systems has been evaluated by FBA, using biomass production as objective function. The biomass equation represents a weighted ratio of the components forming the dry weight of a cell, together with the energy demands for cellular growth and maintenance by ATP hydrolysis. Also, specific determination of a particular organism requires specific measurements of metabolites over pure cell cultures [46], [47], [48]. In the absence of this kind of information for *S. glossinidius*, we adopted the biomass equation defined for *E. coli* K12 as objective function for FBA simulations on the metabolic networks [38]. This equation has been employed for different metabolic networks, and is considered valid when the real biomass composition of the organism under study is unknown [49], [50], [51]. In addition, use of the same biomass equation means the results of FBA simulations on *E. coli* K12 iJR904 and *S. glossinidius* metabolic networks can be compared, thus indicating the relative degree of metabolic independency of *S. glossinidius* with respect to a free-living bacterium, like *E. coli* K12.

The results of FBA simulations in a minimal aerobic environment, with glucose as sole external carbon source, confirm *S. glossinidius'* very recent transition to a host-dependent lifestyle. This is discerned by the complete functionality of its ancestral metabolic network under these minimal conditions at a biomass production rate equivalent to that of a free-living bacterium, like *E. coli* K12. This finding represents novel evidence of its recent transition to a host-dependent lifestyle, together with the lack of co-evolution between *S. glossinidius* and its corresponding *Glossina* host species [52], its ability to be cultured *in vitro* [53], and its genome size, similar to that of free-living bacteria [26]. By contrast, FBA simulations on the *S. glossinidius* extant network reveal that a few gene inactivation events, like those in glycogen biosynthesis and, especially, arginine biosynthesis and *ppc* genes, produce drastic metabolic changes in the bacteria, associated with the evolutionary transition to a host-dependent lifestyle (see Figure 1). The inactivation of the *ppc* gene is particularly relevant because it makes the external supply of L-arginine an essential requirement for functionality of the system. Experimental measurements of Ppc activity in *E. coli* K12, based on ^13^C-labelling experiments and isotopomer distribution measurement by gas chromatography-mass spectrometry reveal that 50.7% of carbon flux in wild-type strains is channelled through this enzyme. Results also show its inactivation triggers the glyoxylate bypass of the TCA cycle, a second anaplerotic pathway that replenishes oxalacetate in response to the Ppc blockage [41], [42]. These results were reproduced in our FBA simulations (see Figure 1). It is also worth highlighting that *ppc* gene is absent in ancient bacterial endosymbionts, as in all *B. aphidicola* strains, in *W. glossinidia* or in *B. floridanus and B. pensylvannicus* [12], [13], [14], [15], [16], [17], [23].

*S. glossinidius* metabolic network robustness to gene deletion events has also been analyzed. Studies into the topology and structure of metabolic systems have concluded that the power-law distribution governing the connectivity of natural networks makes these systems robust to the random removal of nodes, with this being a common organizational property of large-scale network systems, including metabolic networks [54], [55], [56], [57]. Most of these studies focus on topological properties of metabolic networks, analyzing how the random removal of network nodes affects topological parameters like connectivity, or clustering coefficient. However, similar results are obtained when collections of single knockouts are generated for a particular organism, with most single knockouts rendering positive growth on cell cultures [58]. In contrast, the results of single knockout simulations on different metabolic networks presented in this study revealed a progressive decrease of network robustness, with the fraction of lethal knockouts in FBA simulations increasing from 23.6% in a free-living bacterium, like *E. coli* K12, to 55% in *S. glossinidius* extant network (See Figure 2). In fact, this fraction increases to 73% of network genes in the metabolic network of *B. aphidicola* from the pea aphid *A. pisum* [31] and to 71.8% in the metabolic network of *B. aphidicola* from the cedar aphid *C. cedri*. This reflects a common evolutionary trend in bacterial endosymbionts under reductive evolution in terms of a progressive reduction of network robustness associated with different stages of the reductive evolutionary process. The decrease in network robustness is also observed in double gene deletion simulations when considering non-essential genes, increasing the number of synthetic lethal pairs from *E. coli* K12 iJR904 to *S. glossinidius* ancestral and extant networks. This is accompanied by a decrease in the number of reactions catalyzed by isozymes from *E. coli* K12 iJR904 (161 reactions) to *S. glossinidius* ancestral (92 reactions) and extant (39 reactions) networks, with BAps and BCc networks having two and zero reactions catalyzed by isozymes, respectively. A recent study reports that many of these synthetic lethal pairs in *E. coli* are due to isozymes catalyzing essential reactions, and increasing deletion size reveals increased numbers of complex k-connected clusters, thereby indentifying key branching points of the central metabolism [43]. In the present study, the increased number of synthetic lethal pairs combined with the decreased number of isozyme-catalyzed reactions from *E. coli* K12 to the *S. glossinidius* extant network may be because many of the additional synthetic lethal pairs in *S. glossinidius* probably correspond to higher-order synthetic lethals in *E. coli* (e.g., revealed with triple-gene deletion simulations). This indicates that under the same external conditions and with the same biomass equation as objective function, genome reduction decreases the robustness of biological systems to higher-order single gene deletions.

A similar trend was observed by Gabaldon and collaborators on studying the robustness of a hypothetical minimal metabolic system [59], where most mutations had a limited effect on the overall topology of the network but the majority (76%) prevented the biosynthesis of at least one metabolite, equivalent to a biomass constituent on FBA simulations. The fragility of this hypothetical minimal metabolic network, based on a minimal gene repertoire deduced from comparative genomics [60], was highlighted by a stoichiometric analysis. In this case, 49 out of 50 reactions (98%) proved functionally essential. Regarding the ecological association with their insect hosts, the reduction of system robustness to gene deletion events can be associated with the progressive transition to a more stable environment, like that found within host tissues, where environmental fluctuations are less pronounced than in a free-living environment [5], [61]. This would indicate a reduction in the adaptability of the endosymbiotic systems, consequence of the gene inactivation process. This effect would probably be enhanced in advanced stages of the symbiotic association due to the loss of many genes involved in DNA repair pathways [62], [63]. This may even be associated with the tight association of ancient endosymbionts inside bacteriocyte cells with a more stable environment, compared with the wider tissue tropism of bacterial endosymbionts in the initial stages of association, like *S. glossinidius*.

Reductive evolution simulations on the extant metabolic network of *S. glossinidius* render a set of minimal networks able to produce a functional phenotype in terms of biomass production under different external conditions in FBA simulations. The concept of minimal genomes and minimal gene sets is a common issue in evolutionary genomics, arising as soon as the first complete genomes were available for comparison [64], [65], [66]. These computational approaches, based on gene content comparison across different genomes, have been complemented with different experimental studies of gene essentiality in several species [67], [44], [68]. In this context, stoichiometric analysis of metabolic networks by FBA can evaluate to what point a particular metabolic system can be reduced while maintaining a viable phenotype in terms of biomass production. Similar reductive evolution simulations on the *E. coli* K12 iJR904 network, under external conditions simulating the *B. aphidicola* and *W. glossinidia* environment within their corresponding insect hosts, produce minimal gene sets that accurately reproduce their respective gene content [34]. Recently, the role of environment in the emergence and loss of network robustness has been addressed by Soyer and Pfeiffer in a theoretical minimal metabolic system [69], revealing that the fluctuating nature of external conditions is what ultimately defines the emergence of robustness in biological systems. This can be put down to system evolution under fluctuating environments, which increases the number of multifunctional enzymes and redundant pathways. In our simulations, gene and reaction content of minimal networks is strongly dependent on the external conditions under which the system is evolving, in that a nutrient-rich environment is able to generate greater variability in terms of minimal network composition than a nutrient-limited environment. Under minimal aerobic conditions, with glucose and L-arginine as external metabolites, all minimal networks obtained were highly similar, sharing 84.6% of their reactions and 87.4% of their genes. Meanwhile, greater variability was observed under nutrient-rich conditions, with 41 external metabolites available for uptake, with only 60.9% of the reactions and 58.7% of the genes being shared between minimal networks. This suggests that environmental conditions, and the fact that the environment was more or less rich and fluctuant, ultimately define the complexity of the resulting metabolic networks, playing a decisive role in the potential reductive evolution patterns of *S. glossinidius*. Thus, minimal gene sets or minimal genomes must be defined in the context of the environment associated with the organism under study [60].

Open questions for future research are a more accurate profiling of the ancestral network reconstruction and timing of gene loss events once the genomes of closer relatives of *S. glossinidius* are available. The use of *E. coli* K12 as pivotal organism for network reconstruction has the pitfall of the long divergence time between both strains, which can have negative influence in the reconstruction of ancestral metabolic system. This is especially relevant in the identification of gene deletion events, as our parsimony-based approach forcing gene order conservation has the drawback of the rapid loss of gene order in divergent species, enhanced by the high number of rearrangements in bacterial species in initial stages of transition to host-dependent lifestyle [70], [71], [72]. We choose this parsimony-based approach in order to avoid the inclusion of ancestral genes originated from horizontal gene transfer events, but the requirement of gene order conservation make that many ancestral genes in rearranged genome regions lost during *S. glossinidius* evolution are probably missed. In addition, with the current data is not possible to estimate precisely the timing of gene loss and gene inactivation along the evolutionary lineage of *S. glossinidius*, as has been possible in other bacterial lineages where similar parsimony-based approaches has been applied to reconstruct ancestral gene content and timing gene loss events [9], [73], [74], [36]. In this sense, the availability of more closely related genomes to *S. glossinidius* (i.e. different *S. glossinidius* genomes from different *Glossina* species) would allow to identify more precisely the gene loss and pseudogenization events that has took place strictly after the transition to host-dependent lifestyle. Finally, experimental data on *S. glossinidius* cell cultures would also allow to confirm predictions based on the metabolic models and to improve its predictive capability, as has been done with other biological systems [75], [76].

### Conclusions

The reconstruction and functional analysis of the *S. glossinidius* metabolic network at different stages of the genome reduction process reveal how this evolutionary process affects the overall functionality of this bacterium in the context of its association with the tsetse host. The functionality of the ancestral metabolic network under minimal conditions confirms the recent transition of *S. glossinidius* to a host-dependent lifestyle, which can be explained by the drastic changes in the whole metabolic system. These occur due to the inactivation of a few key enzymatic activities, especially the inactivation of the key anaplerotic enzyme phosphoenolpyruvate carboxylase. The comparison with other metabolic networks corresponding to free-living bacteria (*E. coli* K12) and ancient bacterial endosymbionts (*B. aphidicola*) reveals there has been a gradual decrease in network robustness to gene deletion events and to changes in particular enzymatic activities in these bacterial endosymbionts since their initial evolutionary transition to a host-dependent lifestyle. In this scenario, *S. glossinidius* represents the initial stages of this association while *B. aphidicola* represents the more advanced stages, with a deeper integration in the host lifecycle. In addition, reductive evolution simulations on the *S. glossinidius* extant network reveal that the gene and reaction content of minimal networks are strongly dependent on the external conditions under which the system is evolving. Moreover, under nutrient-rich conditions, simulating the tsetse environment, the metabolic system may evolve towards outcomes rather similar to a real minimal genome, like that of *W. glossinidia*. This reflects the potential of network analysis to predict gene content evolution.

## Methods

### Network reconstruction and computational inference of metabolic phenotypes by Flux Balance Analysis (FBA)

**Figure 4:**
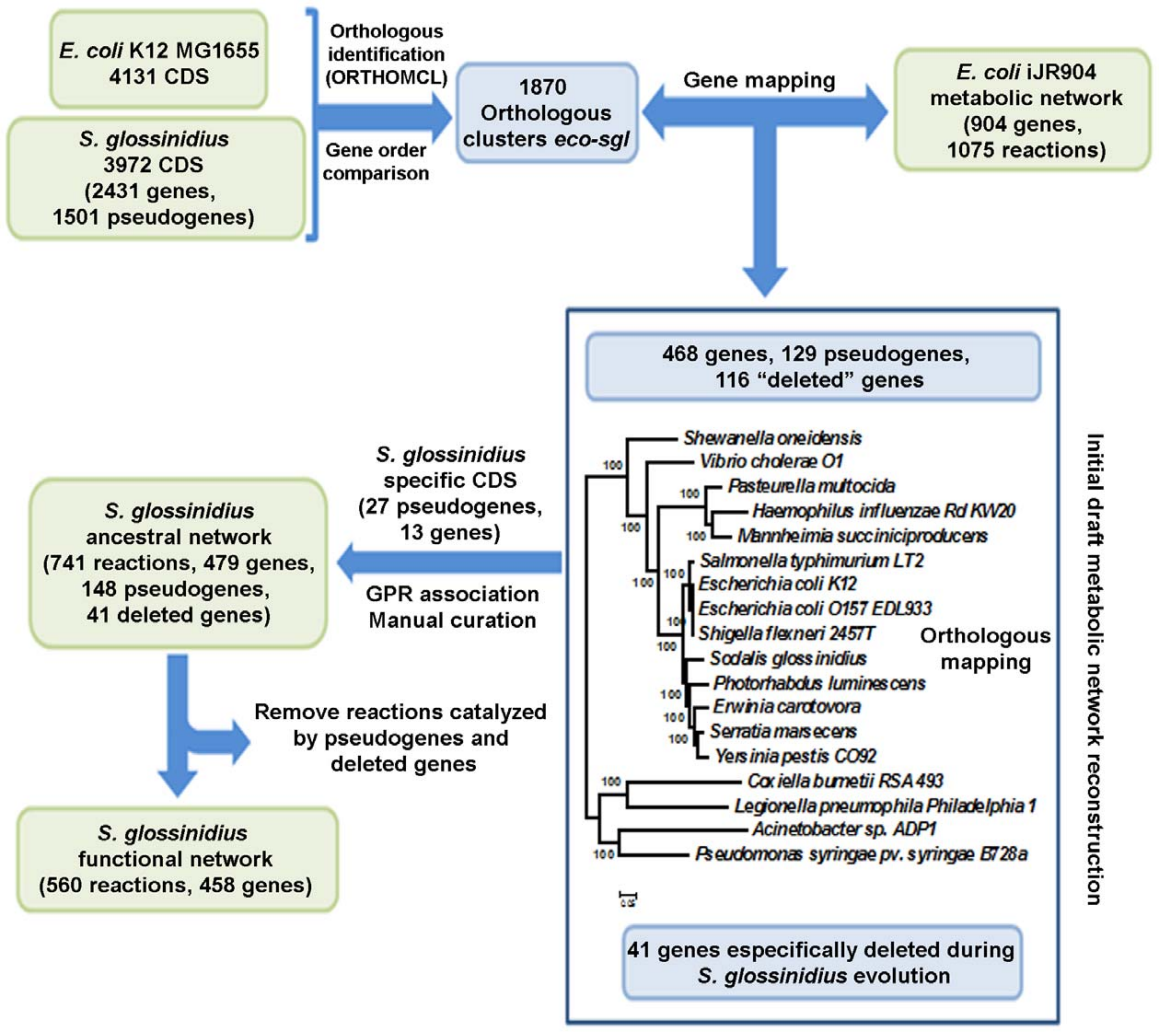
Network reconstruction protocol. Schematic representation of the reconstruction of ancestral and extant genome-scale metabolic networks of *S. glossinidius*. For a detailed explanation of the protocol, see Methods S1.

The network reconstruction process can be broadly divided into three main steps. First, the identification of orthologous clusters between *E. coli* K12 and *S. glossinidius*, which include genes of *E. coli* K12 iJR904 metabolic network, in order to obtain an initial backbone of *S. glossinidius* metabolic networks. Second, a manual curation step to add *S. glossinidius* genes and pseudogenes related with metabolic functions and well defined reaction stoichiometry based on the functional re-annotation of the genome [27], which are not included in the initial mapping with *E. coli* K12. Third, the identification of gene deletion events among the *E. coli* K12 iJR904 network genes in the *S. glossinidius* lineage by comparative genomics. A schematic representation of this process is depicted in Figure 4. For a detailed explanation of the network reconstruction protocol see Methods S1. *S. glossinidius* ancestral and extant metabolic networks in SBML format are provided as Additional Files iEB668.sbml and iEB458.sbml.

Flux Balance Analyses on *S. glossinidius* ancestral and extant metabolic networks were carried out with the COBRA toolbox [77] within the Matlab environment (http://www.mathworks.com/) using the linear optimization algorithm provided by the LP_solve toolkit (http://sourceforge.net/projects/lpsolve/). The biomass equation defined for *E. coli* K12 in their iJR904 reconstruction [38] was used as the objective function to maximize in FBA simulations, in both ancestral and extant metabolic networks of *S. glossinidius*. Maximum oxygen uptake rate was fixed to 20 mmol grDryWeight^−1^ hr^−1^ whereas unconstrained uptake for ammonia, water, phosphate, sulphate, potassium, sodium, iron (II), carbon dioxide and protons was set in concordance with the *E. coli* K12 iJR904 metabolic network. All other external metabolites, except the carbon source, were only allowed to leave the system by fixing the lower bound of their corresponding exchange reactions to zero.

### Robustness analysis

Network robustness of *E. coli* K12 iJR904 and *S. glossinidius* ancestral and extant metabolic networks was evaluated in two different ways. First, the effect of gene deletions over biomass production rates was compared in the metabolic networks of *S. glossinidius* and *E. coli* K12 with the function *singleGeneDeletion* of the COBRA toolbox, limiting the flux over the reactions catalyzed by the deleted gene to zero and calculating biomass production rates in the resulting knockout network. Second, double gene deletion simulations were carried out on non-essential gene pairs, defined in the single knockout simulations with the function *doubleGeneDeletion* of the COBRA toolbox in order to identify synthetic lethal pairs. Non-essential genes are defined as genes with less than 1% of biomass production of the maximum theoretical biomass yield. For comparative purposes, similar single knockout simulations were carried out with two genome-scale metabolic networks of *B. aphidicola*; BAps, corresponding to *B. aphidicola* from the pea aphid *A. pisum* (BAps) [31] and BCc, corresponding to *B. aphidicola* from the cedar aphid *Cinara cedri*, obtained from BAps by removing reactions catalyzed by absent genes in BCc and adjusting the composition of the corresponding biomass equation.

### Reductive evolution simulations

To study the possible future evolution of *S. glossinidius* in the context of the reductive evolutionary process, simulations of reductive evolution were carried out on the *S. glossinidius* extant network to define minimal reactions and gene sets able to support cellular growth in terms of biomass production under different external conditions. The simulations were carried out as follows. Starting with the extant metabolic network of *S. glossinidius*, a randomly chosen reaction was removed from the network by setting the flux through this reaction to zero, and its impact on the metabolic system was evaluated by FBA. If biomass production in the deleted network was above a given cutoff, the reaction was considered as non-essential and was removed permanently, together with its corresponding genes. In contrast, if the biomass production rate over the deleted network was below a given cutoff, the reaction and its corresponding genes were considered essential and were retained in the reduced network. This procedure was repeated progressively until all network reactions were evaluated. Three different cutoffs were considered in terms of the biomass production rate for defining a reaction as essential or non-essential (0.1, 0.01, and 0.05 of the original biomass production rate), and for each cutoff, 500 simulations were carried out. In order to have independent minimal networks in each simulation, the vector of network reactions was randomly permuted in each reductive evolution simulation.

Simulations were performed under two different external conditions in terms of the metabolites available for uptake in FBA simulations. First, nutrient-limited conditions with only glucose and L-arginine as external metabolites, and second, nutrient-rich conditions with 41 external metabolites available for uptake for which the *S. glossinidius* extant network has a functional transport system (See Table S6). These external metabolites were adapted from a previous study by Pal and collaborators [34] where the gene content of *W. glossinidia* was simulated with high accuracy from the *E. coli* K12 iJR904 metabolic network, with external metabolites mimicking the environment of tsetse host tissues. The lower bounds of the corresponding exchange reactions were fixed to −6 mmol gr DryWeight^−1^ hr^−1^ to allow their presence in the extracellular compartment in FBA simulations.

At the end of the simulations, 1500 minimal networks were obtained in each condition, and their corresponding gene content was compared, in order to characterize two different gene sets: i) genes present in all minimal networks in each condition, which can be considered as essential genes for the functionality of the metabolic system; and ii) genes absent in all minimal networks, which can be considered as non-essential or disposable genes that can be always removed without affecting system functionality.

## Supporting Information

Table S1 **Reactions of** ***S. glossinidius*** **ancestral network that includes 27 pseudogenes (highlighted in red) without sequence similarity with** ***E. coli*** **K12.** Pseudogene names follow the notation defined in a recent re-annotation of the genome [27].
(XLS)

Table S2 **Reactions of** ***S. glossinidius*** **extant metabolic network that include genes without sequence similarity with** ***E. coli*** **K12 (highlighted in red).**
(XLS)

Table S3 **Common essential genes and reactions in** ***S. glossinidius*** **minimal metabolic networks under nutrient-limited and nutrient-rich conditions.**
(XLS)

Table S4 **Conditionally essential genes and reactions presents in all minimal networks under nutrient-limited conditions but not under nutrient-rich conditions.**
(XLS)

Table S5 **Genes and reactions absent in all minimal networks under nutrient-limited and nutrient-rich conditions.**
(XLS)

Table S6 **Metabolites available for uptake in reductive evolution simulations under nutrient-rich conditions.**
(XLS)

File S1 ***S. glossinidius*** **ancestral metabolic network in SBML format.**
(SBML)

File S2 ***S. glossinidius*** **extant metabolic network in SBML format.**
(SBML)

Methods S1 N**etwork reconstruction protocol.**
(DOC)
