# Supplementary material for: Metabolic Networks of Sodalis glossinidius: A Systems Biology Approach to Reductive Evolution
Source: PLoS One. 2012 Jan 24;7(1):e30652. doi: 10.1371/journal.pone.0030652 (PMC3265509; doi:10.1371/journal.pone.0030652)
Supplement: Methods S1 — Network reconstruction protocol. (DOC) [file pone.0030652.s009.doc]

**Ortholog identification**

In order to reconstruct *S. glossinidius* metabolic networks, we took *E. coli* K12 as the reference genome because it is the closest free-living relative for which an extensive and completely reconstructed metabolic network is available. The metabolic network of *E. coli* JR904 [1] was used as the reference system for network reconstruction and subsequent metabolic comparison of reconstructed networks. The first step was the identification of orthologous genes between *E. coli* K12 substrain MG1655 (AC:NC_000913) and *S. glossinidius* genes and pseudogenes based on a recent re-annotation of its genome where a total number of 1501 pseudogenes were characterized at both nucleotidic and amino acid level [2]. The program OrthoMCL was used based on BLASTP searches with a maximum e-value cutoff of 10^-20^ and a minimum amino acid identity of 80%. This produced a total set of 2257 orthologous clusters between both genomes, with 169 *E. coli* K12-specific clusters, 193 *S. glossinidius*-specific clusters, and 1895 clusters composed by *E. coli* K12 (genes) and *S. glossinidius* (genes or pseudogenes), of which 68 clusters included more than two genes or pseudogenes corresponding to gene duplicates in one or both genomes. The synteny between *S. glossinidius* and *E. coli* K12 were examined based on TBLASTX comparison of the whole genome sequences followed by manual inspection with Artemis Comparison Tool [3] in order to differentiate truth orthologs from paralog genes created by gene duplication in these 68 clusters, including more than two genes or pseudogenes. Eventually, 1870 clusters of orthologous genes between *S. glossinidius* and *E. coli* K12 were obtained with one gene per genome.

**Network reconstruction of ancestral and functional genome-scale metabolic networks of *S. glossinidius***

In an initial step, 1870 clusters of orthologous genes between *S. glossinidius* and *E. coli* K12 were identified with the OrthoMCL[4] program based on all VS all BLASTP comparisons between both proteomes [5], including *S. glossinidius* genes and pseudogenes characterized in the re-annotation of their genome sequence [2], and analyzing synteny between both genomes in order to differentiate true orthologs from paralogs arising from gene duplication. The 904 genes included in the *E. coli* K12 JR904 network were mapped over the 1870 clusters of orthologous genes between *S. glossinidius* and *E. coli* K12, producing a preliminary set of 597 *S. glossinidius* CDSs (468 genes and 129 pseudogenes) orthologous the to *E. coli* K12 JR904 network genes. In a second step, the 2062 *S. glossinidius* CDSs without orthology with *E. coli* K12 by OrthoMCL were analyzed in order to incorporate genes and pseudogenes encoding enzymes related with metabolic functions for which a complete EC number is associated, allowing the incorporation of 27 pseudogenes and 13 genes to the initial backbone of *S. glossinidius* metabolic networks. Finally, 41 genes deleted during the evolution of *S. glossinidius* were also incorporated to the ancestral metabolic network based on the analysis of the gene context of the 307 genes of E. coli K12 JR904 absent in *S. glossinidius* and their presence in additional γ-proteobacterial genomes.

The result of this analysis was an ancestral set of 481 genes, 156 pseudogenes and 41 deleted genes, which can be considered functional in the ancestral metabolic system of *S. glossinidius*. In order to reconstruct the ancestral metabolic network of *S. glossinidius*, Gene Protein Reaction (GPR) associations were established following the procedure described in Supplementary Methods. This renders a set of 682 reactions in the ancestral network catalyzed by 479 genes, 148 pseudogenes and 41 deleted genes. In addition, 58 reactions without gene association in *E. coli* K12 JR904 network were also included in *S. glossinidius* ancestral network, yielding a final set of 740 reactions in the ancestral metabolic network of *S. glossinidius* (See Additional File iEB668.sbml). In order to define the boundaries of the ancestral metabolic network, 143 exchange reactions included in *E. coli* K12 JR904 network were also included in *S. glossinidius* ancestral network.

*S. glossinidius* functional network was inferred from the ancestral network removing 181 reactions catalyzed by the 148 pseudogenes and 41 deleted genes following the same procedure as described above for the ancestral network, yielding a functional metabolic network composed by 458 genes and 560 internal reactions, that includes the same 143 exchange reactions as the ancestral network to define the boundaries of the system in FBA simulations (See Additional File iEB458.sbml). In the transition to the functional network, 21 genes of the ancestral network were removed because they are associated with enzyme complexes for which at least one of the components is deleted or pseudogenized.

**Incorporation of gene deletion events in the metabolic network of *S. glossinidius***

In order to consider gene deletion events in the metabolic model of S*. glossinidius*, the gene context of the 307 genes of *E. coli* K12 JR904 network without orthology with *S. glossinidius* were analyzed based on whole genome comparisons between both genomes in order to identify syntenic genome regions around these genes. This yields a preliminary set of 116 *E. coli* K12 JR904 network genes absent in *S. glossinidius* but with a conserved genomic context. These genes could have evolved by gene deletion events during *S. glossinidius* evolution or alternatively by horizontal gene transfer events in the lineage leading to *E. coli* K12, in which case they cannot be considered as present in the ancestor of *S. glossinidius*. In order to differentiate between these evolutionary scenarios, these 116 *E. coli* K12 JR904 genes were mapped in the genomes of the related γ-proteobacteria *Serratia marsecens* (<http://www.sanger.ac.uk/Projects/S_marsecens /> ), *Serratia proteomaculans* (NC_009832), *Erwinia carotovora* (NC_004547), *Yersinia pestis* CO92 (NC_003143), *Enterobacter* sp. 638 (NC_004431) and additional *E. coli* genomes of strains O6:H1 CFT073 (NC_004431), O157:H7 EDL933 (NC_002655), and APEC O1 N(NC_008563). The program OrthoMCL was used based on BLASTP searches of all against all proteins with a maximum e-value cutoff of 10^-20^ and a minimum amino acid identity of 80% [4]. Genes that were present in the 4 *E. coli* genomes and at least two additional genomes of γ-proteobacteria were considered as effectively removed during *S. glossinidius* evolution. This analysis yields 41 additional genes of *E. coli* K12 JR904 network specifically deleted during *S. glossinidius* evolution.

**Gene Protein Reaction (GPR) association rules**

An essential step in the process of network reconstruction is the establishment of GPR associations in order to link network reactions to their corresponding enzymes. In this context, based on the initial set of 481 genes, 156 pseudogenes and 41 deleted genes, putatively present in the ancestral metabolic system of *S. glossinidius*, three different types of reactions were considered. First, for reactions catalyzed by a single enzyme encoded by a single gene, if the gene is present as gene, pseudogene or deleted gene the corresponding reaction is included in the ancestral network (520 reactions). Second, for reactions catalyzed by a single enzymatic complex encoded by several genes, if all the corresponding genes are present as gene, pseudogene or deleted genes the corresponding reaction is included in the ancestral network (82 reactions). Third, for reactions catalyzed by different isozymes or isoenzymatic complexes if at least one of the isoforms is present as gene, pseudogene or deleted gene the corresponding reaction is included in the ancestral network (73 reactions).

REFERENCES

1. Reed JL, Vo TD, Schilling CH, Palsson BO: An expanded genome-scale model of Escherichia coli K-12 (iJR904 GSM/GPR). *Genome Biol* 2003, 4:R54.

2. Belda E, Moya A, Bentley S, Silva FJ: Mobile genetic element proliferation and gene inactivation impact over the genome structure and metabolic capabilities of Sodalis glossinidius, the secondary endosymbiont of tsetse flies. *BMC Genomics* 2010, 11:449.

3. Carver T, Berriman M, Tivey A, Patel C, Bohme U, Barrell BG, Parkhill J, Rajandream MA: Artemis and ACT: viewing, annotating and comparing sequences stored in a relational database. *Bioinformatics* 2008, 24:2672-2676.

4. Li L, Stoeckert CJ, Jr., Roos DS: OrthoMCL: identification of ortholog groups for eukaryotic genomes. *Genome Res* 2003, 13:2178-2189.

5. Altschul SF, Madden TL, Schaffer AA, Zhang J, Zhang Z, Miller W, Lipman DJ: Gapped BLAST and PSI-BLAST: a new generation of protein database search programs. *Nucleic Acids Res* 1997, 25:3389-3402.
